# Pre-breeding in alfalfa germplasm develops highly differentiated populations, as revealed by genome-wide microhaplotype markers

**DOI:** 10.1038/s41598-024-84262-x

**Published:** 2025-01-08

**Authors:** Cesar A. Medina, Dongyan Zhao, Meng Lin, Manoj Sapkota, Alexander M. Sandercock, Craig T. Beil, Moira J. Sheehan, Brian M. Irish, Long-Xi Yu, Hari Poudel, Annie Claessens, Virginia Moore, Jamie Crawford, Julie Hansen, Donald Viands, Michael D. Peel, Neal Tilhou, Heathcliffe Riday, E. Charles Brummer, Zhanyou Xu

**Affiliations:** 1 Plant Science Research Unit, USDA-ARS, St. Paul, MN USA; 2 Breeding Insight, Cornell University, Ithaca, NY USA; 3 Plant Germplasm Introduction and Testing Research Unit, USDA-ARS, Prosser, WA USA; 4 Lethbridge Research and Development Center, Agriculture and Agri-Food Canada, Lethbridge, AB Canada; 5 Quebec Research and Development Centre, Agriculture and Agri-Food Canada, Québec, QC Canada; 6 School of Integrative Plant Science, Plant Breeding and Genetics Section, Cornell University, Ithaca, NY USA; 7 Forage and Range Research Unit, USDA-ARS, Logan, UT USA; 8 Dairy Forage Research Center, USDA-ARS, Madison, WI, US USA; 9 Department of Plant Sciences, University of California Davis, Davis, CA USA

**Keywords:** Alfalfa, DArTag, Microhaplotypes, Genetic diversity

## Abstract

Plant genebanks contain large numbers of germplasm accessions that likely harbor useful alleles or genes absent in commercial plant breeding programs. Broadening the genetic base of commercial alfalfa germplasm with these valuable genetic variations can be achieved by screening the extensive genetic diversity in germplasm collections and enabling maximal recombination among selected genotypes. In this study, we assessed the genetic diversity and differentiation of germplasm pools selected in northern U.S. latitudes (USDA Plant Hardiness Zone 7 or below) originating from Eurasian germplasm. The germplasm evaluated included four BASE populations (C0) from different geographical origins (Central Asia, Northeastern Europe, Balkans-Turkey-Black Sea, and Siberia/Mongolia), 20 cycle-one populations (C1) derived from each of the four BASE populations selected across five locations in the U.S. and Canada, and four commercial cultivars. Using a panel of 3,000 Diversity Array Technologies (DArTag) marker loci, we retrieved 2,994 target SNPs and approximately 12,000 microhaplotypes. Microhaplotypes exhibited higher genetic diversity values than target SNPs. Principal component analysis and discriminant analysis of principal components revealed significant population structure among the alfalfa populations based on geographical origin, while the check cultivars formed a central cluster. Inbreeding coefficients (F_IS_) ranged from − 0.1 to 0.006, with 27 out of 28 populations showing negative F_IS_ values, indicating an excess of heterozygotes. Interpopulation genetic distances were calculated using Rho pairwise distances (F_ST_ adapted for autotetraploid species) and analysis of molecular variance (AMOVA) parameters. All BASE populations showed lower Rho values compared to C1 populations and check cultivars. AMOVA revealed that most of the genetic diversity was among individuals within populations, especially in BASE populations (92.7%). This study demonstrates that individual plants in BASE populations possess high genetic diversity, low interpopulation distances, and minimal inbreeding, characteristics that are essential for base-broadening selection. The populations developed in this project serve as valuable sources of novel alleles for North American alfalfa breeding programs, offering breeders access to diverse, regionally adapted pools for improving various alfalfa traits.

**Supplementary Information:**

The online version contains supplementary material available at 10.1038/s41598-024-84262-x.

## Introduction

Genetic diversity is an important characteristic of plant populations because it serves as the starting point for developing improved cultivars. Lack of genetic diversity reduces the ability of breeders to make gains in programs^1^. Plant genbanks acquire, conserve long-term and provide access to genetic diversity and information for plant breeders to use in developing new and improved cultivars. Plant breeders can employ two strategies when utilizing diversity in plant germplasm collections: introgression and incorporation^2^.

Introgression involves crossing and backcrossing to introduce a desired trait into an elite germplasm, typically involving only a small segment of the genome from an exotic germplasm. Introgression focuses on qualitative traits with high heritability, where one or several genes display major effects on the desired traits. In contrast, incorporation involves the development of locally selected populations from exotic germplasm, which can then be integrated into the adapted genetic base in the crop of interest to help improve quantitative traits controlled by multiple genes. Incorporation requires a long-term breeding program to generate locally selected populations concentrating desirable alleles from poorly adapted initial germplasm pools^2^.

Alfalfa (*Medicago sativa* L.) is known as 'the queen of forages’ due to its high productivity, nutritional value as animal feed, and nitrogen-fixing ability. It ranks alongside wheat as the third most economically important crop in the USA^3^. Cultivated alfalfa is an insect pollinated, outcrossing perennial forage legume that is autotetraploid (2n = 4x = 32), exhibits tetrasomic inheritance, and has a haploid genome size of 800 − 1000 Mb^4,5^. Populations are highly heterogeneous and individual genotypes are marked by high levels of heterozygosity. These characteristics provide the crop an abundant allelic variation for adaptation and survival in response to various biotic and abiotic conditions, enabling alfalfa to grow in a wide range of environments^6^. However, progress in increasing alfalfa biomass yields has been stagnant, with a genetic gain of 1% annually over the past 20 years^7^, necessitating the development of new breeding strategies to improve important agronomical traits such as yield.

Genetic bottlenecks and genetic drift can lead to a loss of genetic variability, slowing genetic gain and increasing the risk of crop vulnerability^8^. To avoid genetic vulnerability in alfalfa, breeders create and preserve their own germplasm collections while assessing the current genetic diversity of their programs. For instance, domesticated alfalfa populations from Eurasia and North Africa can be up to 30% less diverse than wild populations from Spain and the Middle East^9^. Further, even in situations where genetic vulnerability is not a concern, rapid environmental changes, such as those increasingly observed under modern human-induced climate change, may create situations where extant genetic variation in breeding programs is not sufficient for optimal adaptation. The U.S. Department of Agriculture, Agricultural Research Service (USDA ARS) National Plant Germplasm System (NPGS) holds 4,083 alfalfa and subordinate taxa accessions^10^ that are sources of diversity for desired traits in commercial alfalfa breeding programs.

Genetic markers have been useful to evaluate population diversity and identify genetic loci associated with traits of interest. For many years, simple sequence repeats (SSRs), co-dominant and multiallelic markers, were widely used for studying genetic diversity or population structure due to their high reproducibility and high frequency throughout genome^11–14^. However, genotyping with SSRs is labor-intensive, and the number of markers used is often limited to fewer than 100. With the development of next generation sequencing methodologies, new high throughput approaches like genotyping by sequencing^15^ or restriction site-associated DNA sequencing^16^ can identify and score thousands of genetic markers randomly distributed across the target genome for large-scale studies.

Recently, a Diversity Array Technology (DArTag) genotyping platform for alfalfa was developed by Breeding Insight^17,18^ that comprises 3,000 target-SNP loci distributed across the alfalfa genome. Each locus is embedded within a sequenced genome region of 54–81 base pairs that are assessed in all samples to 100 × depth. This sequencing identifies closely linked off-target SNPs in addition to the target SNPs. Each short sequence with a unique combination of multiple SNPs is referred to as a microhaplotype^18^. Microhaplotypes can be considered multi-allelic markers, resulting in the potential occurrence of several haplotypes per locus in tetraploids. Therefore, the use of DArTag markers improved the analysis of genetic diversity and added a new and consistent type of marker (microhaplotypes) that combines the advantages of high-throughput genotyping approaches and the resolution power of multiallelic markers.

Collaborators over the years have developed a long-term base-broadening program following the methods outlined by Simmonds (1993) to develop newly selected germplasm populations that could be incorporated into commercial cultivar development programs. For this project, four geographically based germplasm pools to serve as BASE populations were developed by selecting 85 desirable plants —23 for Central Asia (CASIA), 23 for northeastern Europe (EURO), 22 for Balkans-Turkey-Black Sea (Ottoman; OTTM), and 17 Siberia/Mongolia (SIBR) — from a total of 150 germplasm accessions evaluated from northern regions of Eurasia and intercrossing them in isolation^19^.

These pools were then selected at five locations across northern North America —Lethbridge, Alberta (AB); St-Augustin-de-Desmaures, Quebec (QC); Tulelake, California (CA); Ithaca, New York (NY); and Prairie du Sac, Wisconsin (WI)—where desirable plants were again identified and intercrossed by hand in the greenhouse. In this current work, we evaluated the genetic diversity and population structure of 24 alfalfa populations, and four check cultivars (55H94, AmeriStand 427TQ, Hybriforce-4400, and Vernal) using target SNPs and microhaplotypes from the 3 K DArTag platform. We hypothesize that the individually selected populations contain genetic variation distinct from that in commercial breeding germplasm, thus representing potential reservoirs of alleles for future cultivar improvement. The objective of this study was to test this hypothesis by evaluating genetic differences among the initial germplasm pools, among the regionally selected populations, and between the germplasm pools and commercial cultivars.

## Results

### Intrapopulation genetic diversity

**Fig. 1:**
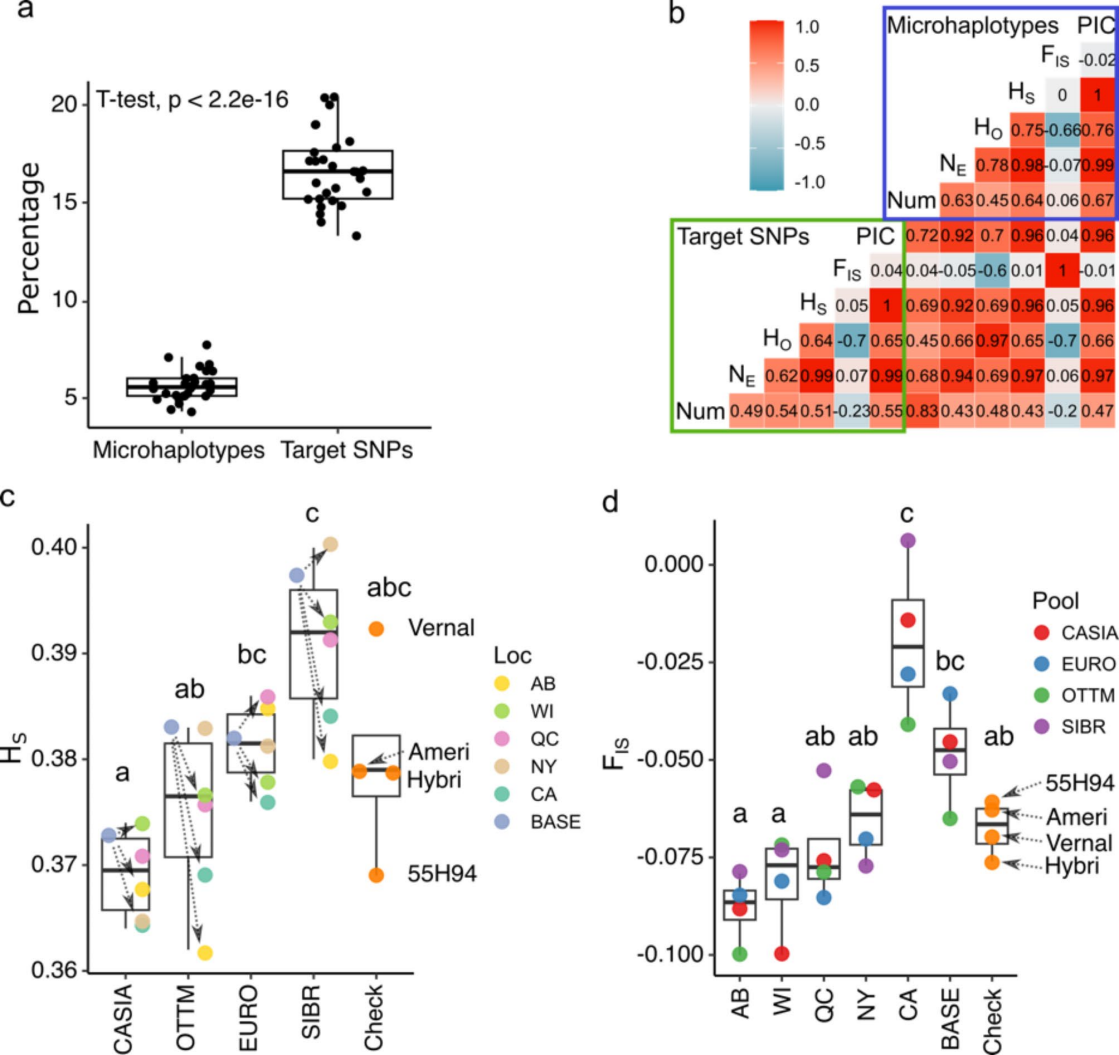
Intrapopulation genetic diversity using target SNPs and microhaplotypes. (**a**) Boxplot for percentage of monomorphic loci in 28 populations using microhaplotypes and target SNPs. (**b**) Pearson’s correlation coefficient between statistics of target SNPs (green box) and microhaplotypes (blue box), including number of alleles (Num), effective number of alleles (N_E_), observed heterozygosity (H_O_), expected heterozygosity within populations (H_S_), inbreeding coefficient (F_IS_), and polymorphism information content (PIC). (**c**) Boxplot for H_S_ values of microhaplotypes grouped by CASIA, OTTM, EURO, SIBR pools or check cultivars (Check). Each point represents H_S_ values colored by locations, and arrows indicate the changes in H_S_ with respect to BASE populations. (**d**) Boxplot for F_IS_ values of microhaplotypes grouped by BASE populations, check cultivars (Check), or by location: Lethbridge, Alberta (AB), St-Augustin-de-Desmaures, Quebec (QC), Tulelake, California (CA), Ithaca, New York (NY), and Prairie du Sac, Wisconsin (WI). Each point represents F_IS_ value colored by genetic pools. Different letters stand for significantly different means (p-value < 0.05) using Tukey’s method for pairwise comparisons. Check cultivars: 55H94, AmeriStand 427TQ (Ameri), Hybriforce-4400 (Hybri), and Vernal.

A total of 2,994 out of the 3,000 marker loci were retained after filtering by missing data. Based on heterozygosity-based statistics, greater genetic diversity was found using microhaplotypes relative to using targeted SNP loci. In total, 12,295 microhaplotypes were discovered in this project, which provided more precise genetic information compared to the 2,994 target SNPs coded as allele dosage (0 to 4). The number of monomorphic loci shared across all populations was 23 for microhaplotypes and 56 for target SNPs (Table 1). The percentage of monomorphic loci was significantly greater (p-value < 0.01) when using target SNPs (mean = 16.6%) compared to microhaplotypes (mean = 5.6%) in the 28 populations (Fig. 1a). On average, the percentage of monomorphic target SNPs in the BASE populations (16%) was less than C1 populations (16.6%), and cultivars (17.4%). Cultivar 55H94 and SIBR-CA had the greatest number of monomorphic loci using target SNPs (20.4% both) while SIBR-NY had the least (13.3%) (Table 1). The intrapopulation genetic diversity parameters, number of alleles (Num), effective number of alleles (N_E_), observed heterozygosity (H_O_), expected heterozygosity within populations (H_S_), and total heterozygosity (H_T_), were significantly greater (p-value < 0.01) when using microhaplotypes compared to target SNPs, as expected. Num was 2.1 times greater in microhaplotypes, while N_E_ was slightly greater in microhaplotypes (1.3 times). H_O_, H_S_, and H_T_ were 1.6, 1.5, and 1.5 times greater, respectively, with microhaplotypes relative to target SNPs (Table 1). Because genetic diversity parameters were slightly more precise using microhaplotypes, the intrapopulation genetic results were summarized only for microhaplotypes.

**Table 1:** Intra-population genetic variability using target SNPs and microhaplotypes. Heterozygosity-based statistics are population size (size), percentage of monomorphic loci (Mon) number of alleles (Num), effective number of alleles (N_E_), observed heterozygosity (H_O_), subpopulation heterozygosity or gene diversity (H_S_), inbreeding coefficient (F_IS_), polymorphism information content (PIC), and total heterozygosity (H_T_).

Info					Target SNPs							Microhaplotypes						
Population	Pool	C	Loc	Size	Mon	Num	*N*_E_	H_O_	H_S_	F_IS_	PIC	Mon	Num	*N*_E_	H_O_	H_S_	F_IS_	PIC
55H94	Check	−	−	38	20.4	1.796	1.404	0.246	0.238	-0.034	0.237	7.1	2.836	1.765	0.391	0.369	-0.061	0.366
AmeriStand 427TQ	Check	−	−	25	19.0	1.810	1.418	0.258	0.248	-0.041	0.246	6.6	2.864	1.776	0.403	0.379	-0.063	0.375
Hybriforce-4400	Check	−	−	54	15.1	1.849	1.418	0.261	0.247	-0.056	0.246	4.9	3.103	1.788	0.408	0.379	-0.076	0.378
Vernal	Check	−	−	48	15.2	1.848	1.438	0.269	0.256	-0.049	0.255	5.2	3.112	1.825	0.419	0.392	-0.070	0.390
CASIA-BASE	CASIA	C0	−	44	16.6	1.834	1.412	0.250	0.245	-0.023	0.243	5.1	3.108	1.766	0.390	0.373	-0.045	0.371
CASIA-AB	CASIA	C1	AB	46	15.5	1.845	1.404	0.258	0.240	-0.072	0.239	5.1	2.991	1.758	0.401	0.368	-0.088	0.367
CASIA-CA	CASIA	C1	CA	32	18.1	1.819	1.406	0.237	0.241	0.019	0.239	6.7	2.841	1.742	0.370	0.364	-0.014	0.362
CASIA-NY	CASIA	C1	NY	48	16.6	1.834	1.400	0.248	0.239	-0.037	0.238	5.5	2.988	1.747	0.387	0.365	-0.058	0.364
CASIA-QC	CASIA	C1	QC	56	15.2	1.848	1.408	0.256	0.242	-0.059	0.241	5.1	3.116	1.766	0.399	0.371	-0.076	0.369
CASIA-WI	CASIA	C1	WI	40	17.8	1.822	1.411	0.264	0.244	-0.082	0.242	6.4	2.908	1.771	0.411	0.374	-0.100	0.371
EURO-BASE	EURO	C0	−	55	14.4	1.856	1.427	0.253	0.251	-0.005	0.250	4.7	3.170	1.792	0.395	0.382	-0.033	0.380
EURO-AB	EURO	C1	AB	40	16.6	1.834	1.428	0.268	0.251	-0.068	0.250	5.7	2.990	1.805	0.418	0.385	-0.085	0.383
EURO-CA	EURO	C1	CA	50	15.5	1.845	1.418	0.245	0.246	0.003	0.245	5.7	3.027	1.776	0.386	0.376	-0.028	0.374
EURO-NY	EURO	C1	NY	58	14.8	1.852	1.423	0.261	0.249	-0.049	0.248	4.9	3.114	1.794	0.408	0.381	-0.070	0.380
EURO-QC	EURO	C1	QC	35	17.1	1.829	1.427	0.268	0.252	-0.065	0.250	5.6	2.924	1.804	0.418	0.386	-0.085	0.383
EURO-WI	EURO	C1	WI	39	16.9	1.831	1.419	0.263	0.248	-0.064	0.246	6.0	2.960	1.779	0.408	0.378	-0.081	0.376
OTTM-BASE	OTTM	C0	−	38	16.0	1.840	1.430	0.264	0.253	-0.043	0.251	5.2	3.107	1.796	0.408	0.383	-0.065	0.381
OTTM-AB	OTTM	C1	AB	39	20.0	1.800	1.396	0.254	0.235	-0.082	0.234	7.7	2.765	1.749	0.398	0.362	-0.100	0.360
OTTM-CA	OTTM	C1	CA	37	19.0	1.810	1.411	0.245	0.242	-0.012	0.241	5.8	2.935	1.760	0.384	0.369	-0.041	0.367
OTTM-NY	OTTM	C1	NY	51	14.0	1.860	1.430	0.262	0.252	-0.036	0.251	4.4	3.255	1.796	0.405	0.383	-0.057	0.381
OTTM-QC	OTTM	C1	QC	46	15.7	1.843	1.418	0.262	0.247	-0.061	0.246	5.4	3.047	1.778	0.406	0.376	-0.079	0.374
OTTM-WI	OTTM	C1	WI	41	17.2	1.828	1.421	0.260	0.248	-0.051	0.246	5.7	3.001	1.777	0.404	0.377	-0.072	0.374
SIBR-BASE	SIBR	C0	−	39	17.1	1.829	1.442	0.263	0.258	-0.021	0.256	5.5	3.141	1.839	0.417	0.397	-0.050	0.394
SIBR-AB	SIBR	C1	AB	51	16.2	1.838	1.420	0.259	0.247	-0.052	0.245	6.0	2.944	1.800	0.410	0.380	-0.079	0.378
SIBR-CA	SIBR	C1	CA	28	20.4	1.796	1.423	0.240	0.249	0.038	0.247	6.4	2.953	1.794	0.382	0.384	0.006	0.380
SIBR-NY	SIBR	C1	NY	57	13.3	1.867	1.443	0.273	0.259	-0.055	0.257	4.3	3.285	1.855	0.431	0.400	-0.077	0.398
SIBR-QC	SIBR	C1	QC	43	17.6	1.824	1.428	0.258	0.251	-0.028	0.250	5.8	3.083	1.827	0.411	0.391	-0.053	0.388
SIBR-WI	SIBR	C1	WI	55	14.8	1.852	1.434	0.266	0.254	-0.049	0.253	5.1	3.102	1.833	0.421	0.393	-0.073	0.391
Total	−	−	−	1,233	0.8	1.982	1.410	0.258	0.248	-0.040	−	1.9	4.107	1.766	0.403	0.379	-0.063	−
H_T_	−	−	−	−	−	−	−	−	0.256	−	−	−	−	−	−	0.389	−	−

Values were calculated for check cultivars (Check), BASE (C0), and cycle one (C1) populations established in five locations (Loc): Lethbridge, Alberta (AB), St-Augustin-de-Desmaures, Quebec (QC), Tulelake, California (CA), Ithaca, New York (NY), and Prairie du Sac, Wisconsin (WI). Size corresponds to the number of plants genotyped.

A negative correlation was observed between H_O_ and F_IS_ (*r* = -0.66) and a strong positive correlation between N_E_, H_O_, H_S_, and PIC values of microhaplotypes (> 0.76) (Fig. 1b). Among those parameters, N_E_ and H_S_ were strongly correlated (*r* = 0.98), indicating the influence of the effective number of alleles on the expected heterozygosity. A comparison of genetic diversity between check cultivars and BASE populations, analyzed as pooled groups, revealed a reduction in Num, N_E_, H_S_, and H_T_ in the cultivar pool compared to the BASE pool, across both microhaplotypes and target SNPs (Supplementary Table 1). H_S_ values were greater in SIBR populations but reduced in CASIA-CA and OTTM-AB populations. 55H94 and Vernal were cultivars with the smallest (0.369) and the greatest (0.392) H_S_ values, respectively. Populations from SIBR were significant different (p-value < 0.05) from CASIA and OTTM pools (Fig. 1c). SIBR-NY was the most diverse population (N_E_ = 1.855 and H_S_ = 0.4) while OTTM-AB was the least diverse population (N_E_ = 1.749 and H_S_ = 0.362). Analysis by location determined that NY was the location with the most diverse populations (N_E_ = 1.798 and H_S_ = 0.382) while CA was the location with the least diverse populations (N_E_ = 1.768 and H_S_ = 0.373) (Table 1).

The inbreeding coefficient (F_IS_) varies depending on the selfing rate and, in autopolyploids, the proportion of double reduction. F_IS_ values range from − 1 to 1, where values ≤ 0 are expected for randomly mating finite populations, and values > 0 indicate a heterozygote deficit. F_IS_ values were assessed with target SNPs and microhaplotypes, and were compared among check cultivars, C0 populations, and C1 populations by location. F_IS_ values were strongly correlated between target SNPs and microhaplotypes (y = -0.029 + 0.85x, R = 1), but the mean was significantly reduced (p-value < 0.05) in microhaplotypes (− 0.06) compared with target SNPs (− 0.04). Most F_IS_ values in microhaplotypes and target SNPs were ≤ 0, (27 and 25 out of 28, respectively). In both cases F_IS_ values were around 0. Among locations, AB had the smallest F_IS_ value (− 0.088) and CA the greatest (− 0.019). All CA populations exhibited increased F_IS_ in comparison with BASE populations (0.005 in EURO to 0.055 in SIBR). F_IS_ mean value in populations from CA were significant different (p-value < 0.05) from other locations and cultivars (Fig. 1d). Inbreeding coefficients in cultivars were similar, ranging from − 0.076 in Hybriforce-4400 to − 0.061 in 55H94 (Table 1).

### Interpopulation distances

Genetic differentiation among populations was assessed using F_ST_ and Rho parameters using target SNPs and microhaplotypes (Supplementary Tables 2 and 3). F_ST_ is a measure of genetic differentiation among populations on a scale from 0 to 1, where 0 indicates no differentiation and 1 represents complete differentiation. Rho is a similar measure, adjusted to account for ploidy level. Strong concordance was observed between pairwise F_ST_ (y = 0.0012 + 0.81x, R = 0.99) or Rho (y = 0.0038 + 0.9x, R = 0.99) values using target SNPs and microhaplotypes (Supplementary Fig. 1a). Similarly, a strong positive linear relationship was observed between target SNPs (y = 0.012 + 3.7x, R = 0.99) and microhaplotypes (y = 0.0098 + 4.1x, R = 0.99) using F_ST_ and Rho pairwise values (Supplementary Fig. 1b).

**Fig. 2:**
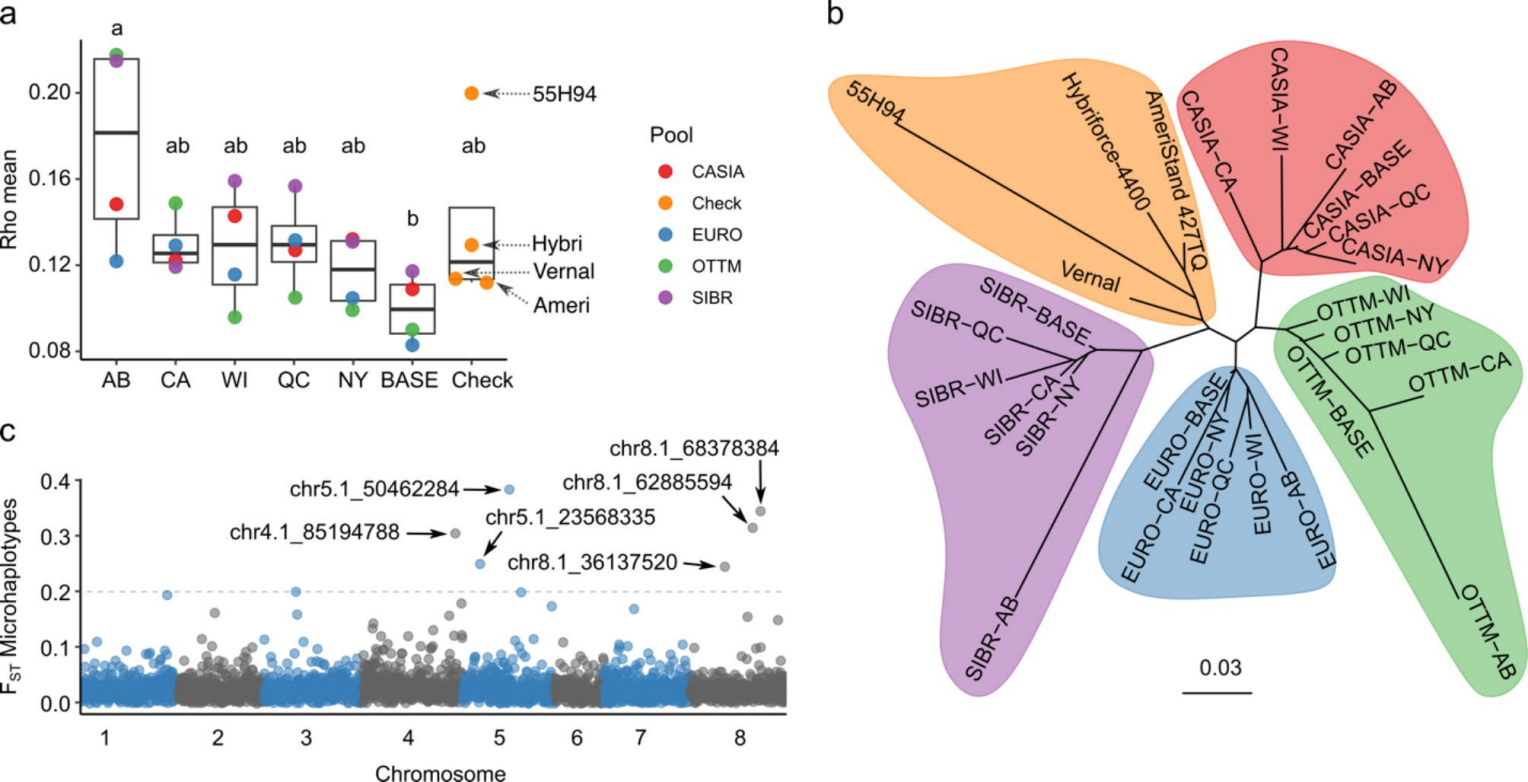
Genetic differentiation in alfalfa populations. (**a**) Boxplot for mean Rho values of 28 alfalfa populations using target SNPs. Different letters stand for significantly different means of locations (p-value < 0.05) using Tukey’s method for pairwise comparisons. (**b**) Neighbor-joining tree among alfalfa populations based on Rho pairwise distance matrix. The unrooted tree identifies five clusters according to defined germplasm pools. The bar corresponds to the scale edge length. (**c**) Locus-specific estimates of F_ST_ in 28 alfalfa populations using microhaplotypes. Markers listed in the plot have genetic differentiation (F_ST_) > 0.2. Twenty-eight alfalfa populations correspond to four check cultivars (55H94, AmeriStand 427TQ (Ameri), Hybriforce-4400 (Hybri), and Vernal), four BASE (C0), and 20 cycle-one (C1) populations established in five locations (Loc): Lethbridge, Alberta (AB), St-Augustin-de-Desmaures, Quebec (QC), Tulelake, California (CA), Ithaca, New York (NY), and Prairie du Sac, Wisconsin (WI).

Rho pairwise distances calculated for 28 alfalfa populations using target SNPs had greater genetic differentiation values (0.131) compared to microhaplotypes (0.122) (Supplementary Table 3). Rho pairwise values ranged from 0.007 to 0.336. The mean Rho values were lower in BASE populations (0.1) compared to C1 populations (0.136) and check cultivars (0.139) (Fig. 2a). Populations SIBR-BASE and SIBR-NY were most similar (Rho = 0.007), and population OTTM-AB and SIBR-AB were most different (Rho = 0.336). Compared to other populations OTTM-AB, SIBR-AB, and cultivar 55H94 have the greatest mean Rho values of 0.218, 0.215, and 0.200, respectively. Genetic differentiation was larger in AB with the greatest Rho values in OTTM, and SIBR populations. The neighbor-joining tree based on the Rho pairwise distance matrix showed that these populations were primarily structured according to germplasm pools (CASIA, EURO, OTTM, SIBR, and check cultivars). OTTM-AB, SIBR-AB, and 55H94 were the most distant populations from their pools with the largest edge lengths (Fig. 2b).

Genetic differentiation by locus was determined using F_ST_ values from microhaplotypes. F_ST_ values ranged from 0 to 0.383 with a mean value of 0.026 (Fig. 2c). Six loci showed large or very large effect (F_ST_ values > 0.2), indicating their influence on genetic differentiation among populations, two of those loci contained private alleles (Fig. 2c). Private alleles were defined as alleles found exclusively in one population with a frequency > 0.05. For instance, haplotype 3 in the chr4.1_85194788 locus was a private allele of the cultivar 55H94, with a frequency of 0.38 (Supplementary Table 4). Other loci with elevated F_ST_ values harbor interesting alleles. For example, haplotype 3 in the chr8.1_62885594 locus (F_ST_ = 0.314) has greater allele frequency in the SIBR populations (0.38) compared to other populations (0.01).

Out of 12,295 microhaplotypes, 811 (6.6%) were private alleles. The frequency of private alleles ranged from 0.051 to 0.480 (Supplementary Fig. 2a). Nine private alleles had a frequency > 20% and were found in SIBR-AB and cultivar 55H94 (Supplementary Fig. 2b). cultivar 55H94 (145), SIBR-AB (104), and OTTM-AB (59) exhibited the greatest number of private alleles, while BASE populations had 28 private alleles.

The Analysis of Molecular Variance (AMOVA) of all 28 populations revealed a percentage of variance among populations of 12.4%, with the rest of the variation within populations. The AMOVA of BASE populations indicated that most genetic variance is among individuals within populations (92.7%) and a small percentage of variance AP (7.3%). The variation among BASE populations (7.3%) was less than among check cultivars (10.6%). Finally, a greater genetic differentiation (11.3% − 21.9%) was observed in C1 populations grouped by location compared with BASE populations. The variation among C1 populations was moderate in NY (11.3%), CA (13.3%), WI (14%), and QC (14.4%), and large in AB (21.9%). All differences among populations were significant (p < 0.001) after 1,000 random permutations to ensure accuracy in the percentage of total variance (Table 2).

**Table 2:** Hierarchical Analysis of Molecular Variance (AMOVA) for all 28 populations or grouped in BASE populations, check cultivars (Check), or by location: Lethbridge, Alberta (AB), St-Augustin-de-Desmaures, Quebec (QC), Tulelake, California (CA), Ithaca, New York (NY), and Prairie du Sac, Wisconsin (WI).

Source	d.f.	SS	MS	V-comp	%Var
All					
AI/WP	1,205	275,087	228	228	87.6
AP	27	44,554	1,650	32	12.4
BASE					
AI/WP	172	42,324	246	246	92.7
AP	3	3,293	1,098	20	7.3
Check					
AI/WP	161	36,195	225	225	89.4
AP	3	3,900	1,300	27	10.6
AB					
AI/WP	172	35,387	206	206	78.1
AP	3	8,188	2,729	58	21.9
CA					
AI/WP	143	37,280	261	261	86.7
AP	3	5,112	1,704	40	13.3
NY					
AI/WP	210	48,098	229	229	88.7
AP	3	5,366	1,789	29	11.3
QC					
AI/WP	176	38,949	221	221	85.6
AP	3	5,663	1,888	37	14.4
WI					
AI/WP	171	36,854	216	216	86
AP	3	5,202	1,734	35	14

The source of variation was among individuals within populations (AI/WP) and split among populations (AP). SS is the sum of squares, MS is the expected mean squares, Var-comp is the component of variance and %Var is the percentage of variance attributed to this source. All differences were significant (p < 0.001) after 1,000 random permutations to obtain accuracy on the percentage of total variance.

### Population structure

**Fig. 3:**
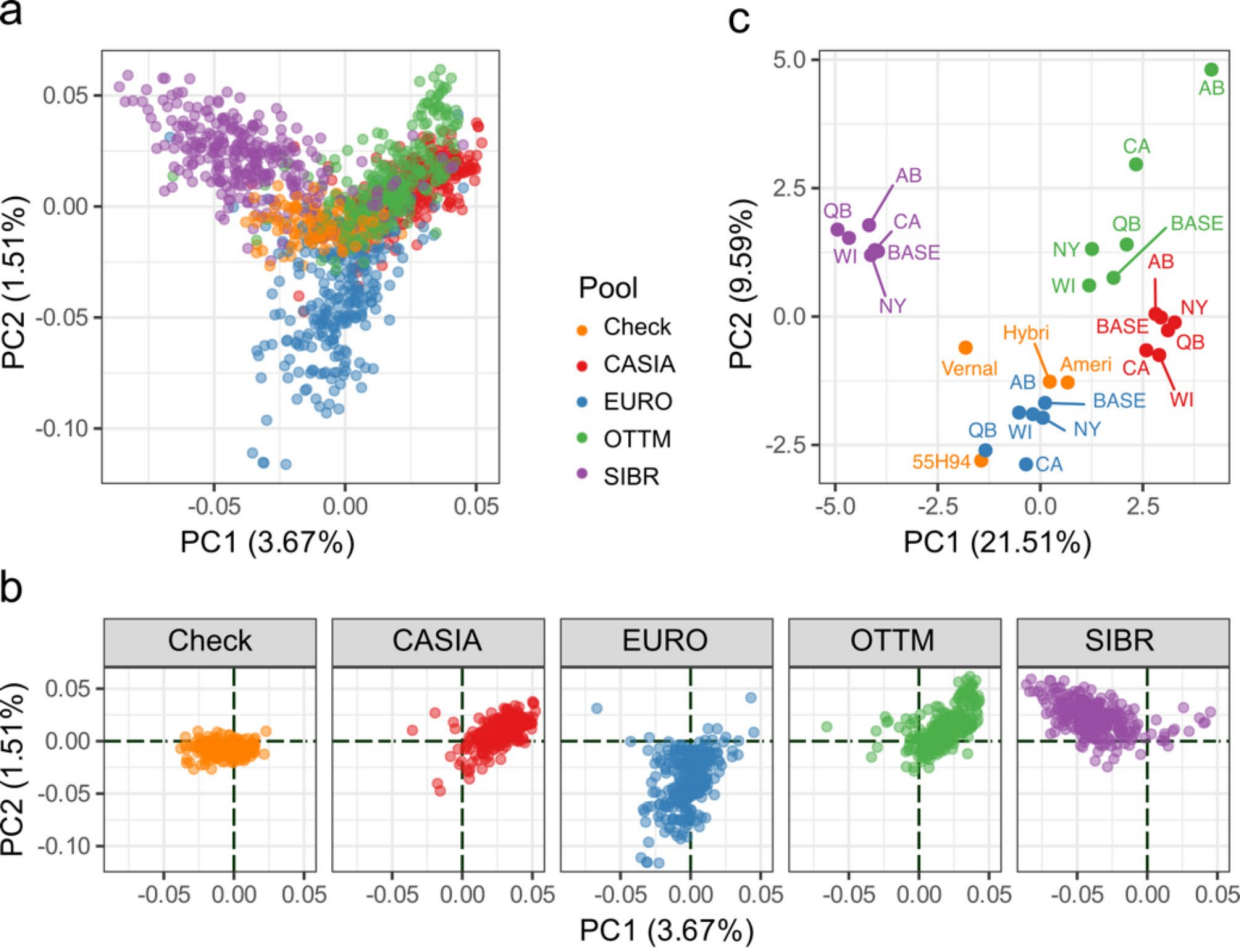
Population structure using Principal Component Analysis (PCA). (**a**) Two-dimensional scatter plot of PC1 and PC2 for 1,233 plants genotyped with target SNPs. (**b**) Sub-plots of the PCA scatter plot colored by germplasm pools or check cultivars (Check). Black dashed lines correspond to coordinate 0 in x and y axis. (**c**) Two-dimensional scatter plot obtained from PCA of 1,233 plants genotyped clustered in 28 defined populations genotyped with 2,994 target SNPs. Samples were colored by germplasm pools or check cultivars (Check). Check corresponds to four check cultivars: 55H94, AmeriStand 427TQ (Ameri), Hybriforce-4400 (Hybri), and Vernal.

A principal component analysis (PCA) was conducted for 1,233 individuals to define the genetic relationships among and within pools using 2,994 target SNPs. The first and second components collectively contributed 5.18% of the total genetic variance, representing 3.67% and 1.51% of the total variation, respectively. In the PCA by plants, genotypes were clustered according to the specific genetic backgrounds (Fig. 3a). Check cultivars were positioned in the middle of the PCA plot, CASIA and OTTM populations in the upper-right part of the plot, the SIBR populations in the upper-left part, and EURO in the lower part (Fig. 3b). There were no differences in pool clustering among locations (Supplementary Fig. 3). Genotypes of individual plants were grouped by populations, and a PCA was conducted to identify clusters by pool. The first and second components explained 21.51% and 9.59% of the total variance. SIBR populations were in the upper-left part of the plot, EURO populations in the lower part, and CASIA populations in the right part, separated from OTTM populations, and check cultivars were spread and close to EURO populations (Fig. 3c).

Because the low percentage of the total genetic explained variance by the two first components, discriminant analysis of principal components (DAPC) was implemented to corroborate the genotype clustering according to the specific genetic backgrounds. DAPC was useful for assigning plants to specific clusters. Around 500 PCs were included in the preliminary step of data transformation and allowed the DAPC to explain 80% of the total genetic variation (Supplementary Fig. 4a). Cluster one exclusively comprised plants of the cultivar 55H94 while the rest of the commercial cultivars were grouped into clusters one, two, and three (Supplementary Table 5). Cluster two gathered populations from different origins, mainly of EURO plants (62.3%), followed by OTTM plants (10%), CASIA plants (8%), and SIBR plants (6.6%). Additionally, cluster two included all plants of Vernal, eight plants of Hybriforce-4400, and one plant of 55H94. Cluster three comprised OTTM plants (73.3%), all plants of AmeriStand 427TQ, and 46 out of 54 plants of Hybriforce-4400. Clusters four and five were mainly composed of CASIA and SIBR plants, accounting for 97.9% and 96.4%, respectively (Supplementary Fig. 4b and Table 5).

### Linkage disequilibrium decay

**Fig. 4:**
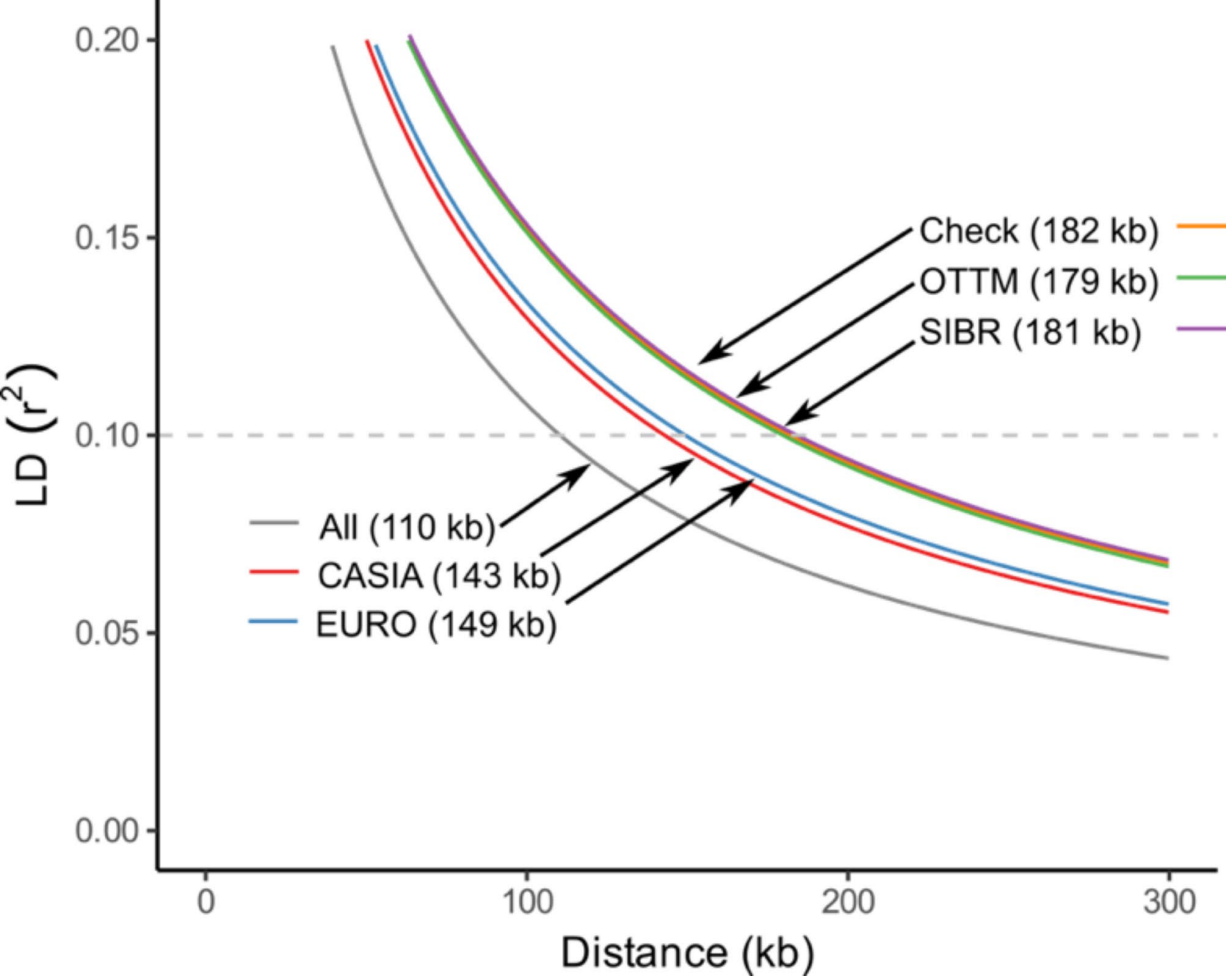
Genome-wide linkage disequilibrium (LD) decay using target SNPs. LD decay was calculated for all genotypes (1,233 plants) and recalculated by genetic pool using 266 plants for CASIA, 277 for EURO, 252 for OTTM, 273 for SIBR, and 165 for check cultivars. The legend contains the LD decay distance in kb at $\:{r}^{2}=0.1$.

The magnitude of linkage disequilibrium (LD) and its decay determine the optimal number of SNPs for association studies and reveals the recombination history within populations. LD decay was estimated by fitting a non-linear model between the correlation coefficient ${(r}^{2})$ among pairs of SNPs and physical distances on the *M. sativa* genome (kb). Across all genotypes (1,233 plants), LD decay at *r*^*2*^ = 0.1 was the shortest (110 kb), with values increasing when calculated using plants from the same breeding pool. Check cultivars (165 plants), OTTM (252 plants), and SIBR (273 plants) exhibited similar LD decay (~ 180 kb), while CASIA (266 plants) and EURO (277 plants) showed a shorter LD decay (~ 145 kb) (Fig. 4).

## Discussion

To breed alfalfa cultivars that are adapted to and perform well in future environments, breeders should leverage the genetic diversity held in gene banks to develop new broad-based populations that potentially carry alleles (and genes) not present in existing breeding programs^20^. While evaluating individual germplasm accessions for traits such as disease resistance is an obvious use of germplasm collections, a potentially more impactful approach for the long-term improvement of the crop is to develop broad-based populations from large numbers of accessions for use in breeding programs^2^.

The current study compared the genetic diversity and population structure within and among four cycle zero populations, 20 regionally selected populations, and four commercial cultivars. In this work, we refer to all these as 28 alfalfa populations. Our results suggest that the plant introduction germplasm accessions from the NPGS collection contain genetic diversity not present in the four commercial cultivars of elite North American breeding pools tested here. Therefore, breeding and selecting from these exotic accessions could generate new germplasm, which could be integrated into the narrow genetic base of elite germplasm using a bridging strategy^21^.

### Target SNPs vs microhaplotypes

Different molecular markers have been employed to study genetic diversity in alfalfa. Among them, simple sequence repeats have proven to be one of the most extensively used markers in tetraploid alfalfa for molecular genetic research^11–14^, but their use have decline with the advent of high-throughput sequencing. On the other hand, SNPs are the most common molecular markers, but they have the lowest allelic richness, resulting in the lowest parameters for genetic structure^22,23^.

During the development of the DArTag genotyping platform in alfalfa, Zhao et al. (2003) highlighted the possibility of using off-target SNPs within 54–81 bp regions harboring target SNPs to identify microhaplotypes^18^. Here, we identified 12,295 microhaplotypes generated during sequencing of the original 2,994 DArTag loci. In our work, the total allele count increased 2.1 times, and the number of monomorphic loci was reduced 2.4 times in microhaplotypes compared to target SNPs. Multiple metrics for intra- and inter-genetic diversity showed improved resolution using microhaplotypes.

The microhaplotypes from the DArTag platform increase the current resources of molecular markers in alfalfa, facilitating investigations for genetic diversity analysis, conservation, marker-trait association, and quantitative trait loci mapping in alfalfa. While several studies have employed marker-based estimation of genetic diversity and population structure in alfalfa, most of them are limited in the number of accessions included and/or the number of markers used to characterize genetic diversity^11,14,24^. The implementation of genotyping by sequencing (GBS) has increased the number of SNPs available for analyzing genetic diversity in alfalfa^22,23,25^. However, SNP markers generated through GBS can be affected by the choice of restriction enzyme, bioinformatic pipeline, or filtering parameters, making it challenging to maintain marker consistency across different GBS projects. In this work, the DArTag platform was used to increase the number and the consistency of markers used to characterize genetic diversity and population structure in the development of regionally selected alfalfa germplasm populations.

### Intrapopulation genetic diversity

BASE populations exhibited the lowest percentage of monomorphic loci (16%) compared to cycle one populations (16.6%) or cultivars (17.4%). Additionally, the mean H_S_ values were greater in BASE populations (0.384) compared to C1 populations (0.378) and alfalfa cultivars (0.380), indicating a slight reduction in genetic diversity following random mating and selection across different locations. Notably, there is a strong negative correlation (R =  − 0.83) between the percentage of monomorphic loci and population size.

Compared to diploid populations, polyploid populations can harbor more genetic diversity because the greater number of chromosome homologous reduces the impact of genetic drift and mutation rates^26^. The most widely used index to measure genetic diversity in a population is expected heterozygosity (H_S_) also known as gene diversity^27^. Genetic diversity in alfalfa germplasm has been consistently high in various studies using SSR markers. Flajoulot et al. (2005) found genetic diversity ranging from 0.665 to 0.717, Bagavathiannan et al. (2010) reported a range from 0.73 to 0.77, and Qiang et al. (2015) observed genetic diversity from 0.629 to 0.762 in the germplasm analyzed^14^. Our H_S_ results, which ranged from 0.235 to 0.259 with target SNPs and 0.362 to 0.4 with microhaplotypes, were lower than previous reports in alfalfa using SSRs. However, we included all loci, while the previous reports filtered the SSRs by a minor allele frequency > 0.05 keeping only diverse markers^11^.

There are interesting findings when comparing the 28 populations individually. Genotypes selected from germplasm pools provide the starting point for genetic diversity in BASE populations. Subsequently, BASE populations, selection, and environmental factors influenced the genetic diversity of C1 populations. Here, we observed that the SIBR-BASE population had high H_S_, and C1 populations bred in NY, QC, and WI showed similar values. However, AB and CA populations exhibited a reduction in H_S_. California C1 populations consistently showed a reduction in H_S_ compared to the BASE populations and an increase in the inbreeding coefficient.

In this study, we included four commercial cultivars from different breeding programs, each representing alfalfa diversity in North America. Vernal is an old public cultivar developed by the University of Wisconsin in 1953, best suited to northern climates^28^. 55H94 developed by DuPont Pioneer in 2011, is resistant to potato leafhopper^29^. AmeriStand 427TQ, released by America’s Alfalfa in 2014, is known for its high yield and fast recovery^30^. Finally, Hybriforce-4400 is a hybrid alfalfa cultivar developed by Dairyland Seed released in 2020 with improved yield potential^31^. Genetic diversity in cultivars ranged from 0.369 in 55H94 to 0.392 in Vernal, and these values were similar to C1 populations. This result agrees with previous results from Flajoulot et al. (2005) where the H_S_ of seven cultivars were similar to a breeding pool of Flemish landraces^11^. Interestingly, while 55H94 is a relatively new cultivar and Vernal is an older public cultivar, genetic diversity may be influenced by factors other than the year of release, such as winter hardiness or resistance to biotic stress.

The accumulation of favorable additive alleles is the primary type of gene action responsible for improved performance in alfalfa^32^. However, during the generation of new populations, there is a chance of mating between relatives if the BASE population is small, leading to inbreeding. Alfalfa has severe inbreeding depression caused by increased homozygosity at loci with deleterious recessive alleles or decreased heterozygosity at loci displaying a heterozygous advantage^33^. In this work, we collected data on the inbreeding coefficient (F_IS_) as a metric of nonrandom mating. A population with F_IS_ = 0 means that individuals are mating randomly, and H_O_ is equal to H_S_. Positive F_IS_ (H_O_ < H_S_) indicates that individuals are experiencing either mating with close relatives or self-fertilization. Negative F_IS_ (H_O_ > H_S_) indicates an excess of observed heterozygotes and occurs due to the presence of non-random mating in genetically distinct individuals^34^. All populations studied here have an excess of observed heterozygotes which means they were not generated by mating with close relatives or by self-fertilization and the death of plantlets was not caused by inbreeding.

### Interpopulation genetic diversity

Genetic differentiation among populations was calculated using fixation index (F_ST_), Rho, and AMOVA. According to different authors, Rho is a parameter designed for polyploids that corrects the ploidy effect to measure genetic differentiation and should be included in diversity studies for polyploids (see formulas 1 and 2 in materials and methods)^26,35^. In alfalfa, Flajoulot et al. (2005) found low but significant F_ST_ values ranging from 0 to 0.012, but these values were 3.6 times greater when genetic differentiation was measured with the Rho parameter^11^. Here we reported that F_ST_ underestimates genetic differentiation in alfalfa, with values 1.12 times lower compared to Rho and our genetic differentiation results are greater than those reported by other authors^25,36^.

BASE populations were generated by intermating selected plants out of accessions from four different regions. Two of these regions are considered as the putative centers of diversity and origin for alfalfa (OTTM and CASIA)^37^, and two regions are areas where alfalfa was subsequently introduced (EURO and SIBR). According to Russelle (2001), alfalfa was introduced into Greece from the OTTM region around 490 B.C., and then it may have spread throughout Europe via the Roman Empire. On the other hand, alfalfa from Uzbekistan (CASIA) was transported (indirectly) to China around 100 B.C.^38^ with low genetic interchange with the SIBR pool. Our results show that SIBR and CASIA pools have low genetic flow (Rho = 0.136), but somewhat surprisingly, SIBR and EURO are more related (Rho = 0.086) which suggest a genetic flow between these two regions. Additionally, *Medicago sativa* ssp. *falcata* (ssp. *falcata*) shares geographical distribution with the OTTM, SIBR, and EURO pools, making it possible that ssp. *falcata* crossed with these pools and introduced new allelic diversity. Introgression from *Medicago sativa* ssp. *falcata* to *Medicago sativa* ssp. *sativa* has been reported to brought frost resistance and enrich the gene pool by introducing approximately 9.9% of climate-adapted genomic regions and stress-resistance genes^6,39,40^.

An AMOVA was conducted to partition molecular genetic variance within and among populations. These analyses, which included all genotypes split the genetic variation into 12.4% among populations and 87.6% within populations. This represents a greater proportion of the variation accounted for by differentiation among populations compared to previous reports^14,36^. Alfalfa cultivars exhibited the highest variation among populations (i.e., among check cultivars) (10.6%), while BASE populations had the lowest variation among populations (7.3%). These results are important because low genetic variation among populations is common in panmictic populations and is associated with extensive recombination, weak selection, and minimal inbreeding, factors that are particularly relevant for base-broadening program.

Populations clustered by location exhibit a larger percentage of among populations variation compared to BASE populations. This result is attributed to assortative mating and biotic and abiotic effects during the development of regionally selected populations. The five locations used to develop regionally selected populations can be classified according to the plant hardiness zone map. This map establishes zones based on the average annual extreme minimum winter temperature, displayed in 10-degree Fahrenheit zones and 5-degree Fahrenheit half zones half zones^41^. Lethbridge, Alberta (AB) is in zone 4b (− 31.7 to − 28.9ºC), St-Augustin-de-Desmaures, Quebec (QC) and Prairie du Sac, Wisconsin (WI) belongs to zone 5a (− 28.9 to − 26.1ºC), Ithaca, New York (NY) is in zone 6a (− 23.3 to − 20.6ºC), and Tulelake, California (CA) is in zone 6b (− 20.6 to − 17.8ºC). AB location has the greatest among populations variation (21.9%). AB experiences the harshest winters, which may be selecting against non-selected germplasm due to insufficient winter hardiness or lack of fall dormancy. Different numbers of plants selected and intercrossed and different contributions of plants to the pollen pool during intercrossing could also affect the population’s genetic parameters. Other factors such as pests and diseases could be influencing the selection of germplasm even further.

Private alleles provide information about the uniqueness of these found in a population, which can be exploited in breeding programs. In this study, we reported that 6.6% of alleles were private alleles in the populations studied. While there are no previous reports of private alleles being identified in alfalfa populations, this percentage is relatively low compared to other crops, such as *Brachiaria* grass. In a study on *Brachiaria* the authors genotyped 79 ecotypes from Kenia using 22 SSRs, reporting that 25 (21%) out of 120 different alleles were private alleles^42^. In this study, private alleles were frequent in cultivar 55H94 and rare in the BASE populations, which may be related to selection process during the cultivar development or genetic drift^43^.

### Population structure

Principal Component Analysis (PCA) and Discriminant Analysis of Principal Components (DAPC) are useful approaches to visualize the genetic structure and gain deeper insights into relationships among populations or genetic pools. In our study, we applied PCA to target SNPs and microhaplotypes, revealing no substantial differences in population structure between marker types, indicating the robustness of the population structure analysis. Using SNPs on 1,233 individual plants it was clear that clusters corresponded to the different germplasm pools. Notably, populations from Central Asia (CASIA) and the Balkan-Black Sea (OTTM) regions overlapped in the two-dimensional scatter plot of PCA by individual plants. Those regions have been considered the center of origin for alfalfa^37^. Here the PCA results and a low Rho value between BASE-CASIA and BASE-OTTM (0.06), indicates a genetic flow between these two regions. PCA by populations had high genetic variation explained by the first two principal components in comparison with PCA by plants. This analysis revealed a clear differentiation of the SIBR populations from the others, likely attributed to a different genetic background, caused by the introgression with *Medicago sativa* ssp. *falcata*^44^.

DAPC is a multivariate statistical approach used to infer the optimum number of clusters of genetically related individuals^45^. Like PCA, DAPC revealed five well-defined clusters separating individuals based on geographical region. However, DAPC splits the check cultivars into three clusters: cluster one, exclusively consists of plants of 55H94, cluster two with Vernal, and cluster three with Hybriforce-4400 and AmeriStand 427TQ. While PCA and DAPC results agree in identifying population structure, the latter technique distinguishes 55H94 from the rest of the alfalfa cultivars. This observation aligns with the results of private alleles and interpopulation analysis.

### Linkage disequilibrium

LD decay helps determine the marker density necessary for association mapping analyses and is also useful for identifying selection pressure among populations^46^. Additionally, LD decay provides information about population response to both natural and artificial selection, reflecting the geographic subdivision and breeding system. For example, population isolation and breeding can result in much longer LD block lengths within a population^47^. Natural populations typically exhibit high recombination rates and low LD decay. However, breeding cycles can reduce diversity and increase LD block size^48^. The LD block size was smallest when all genotypes were included (110 kb), followed by CASIA (143 kb), EURO (149 kb), OTTM (179 kb), and SIBR (181 kb). The extended LD in OTTM and SIBR likely reflects a bottleneck effect during the breeding process^49^, while the shorter LD decay in CASIA and EURO pools suggests random mating, with recombination and independent assortment between loci. LD decay with values around 100 kb agree with previous findings for breeding populations genotyped with DArTag and SNP arrays^48,50^. This information will be valuable in future association mapping studies to help define the LD block size needed to identify candidate genes.

### Conclusion and remark

This is the first report to utilize microhaplotypes in genetic diversity studies, demonstrating that these markers combine the advantages of high-throughput genotyping approaches like GBS with the resolution power of multiallelic markers such as SSRs. Our findings show that microhaplotypes exhibit higher values of both intra- and inter-population diversity compared to target SNP markers. Implementing microhaplotypes for genetic diversity studies does not incur additional costs for DArTag technology, but it increases allelic diversity and enhances the likelihood of identifying private alleles. We found that BASE populations has greater genetic diversity and lower interpopulation structure values compared to cycle one populations, particularly those selected in Alberta and California. Additionally, we were able to differentiate and cluster populations among geographical pools. Our results consistently highlight the cultivar 55H94 as a distinct population, with a large number of private alleles, a high mean Rho value, and an exclusive cluster by DAPC. The populations developed in this project serve as sources of novel alleles for alfalfa breeding programs in North America, introducing new genetic diversity from four different regions of Eurasia. Increasing breeder access to diverse, yet regionally selected pools could lead to breakthroughs in improving currently stagnant alfalfa yields.

## Materials and methods

### Plant materials

Four cycle zero (C0) or BASE populations were initially developed in Wisconsin by selecting 85 agronomically superior plants from 150 NPGS plant introduction (PI) germplasm accessions that originated from four geographically defined Eurasian regions: Central Asia (CASIA) (23 PIs), northeastern Europe (EURO) (23 PIs), Balkans-Turkey-Black Sea (Ottoman; OTTM) (22 PIs), and Siberia/Mongolia (SIBR) (17 PIs) regions (Supplementary Fig. 5a)^51^. The BASE populations were used to develop cycle one (C1) populations at five northern latitude North American locations to select for persistence, superior plant vigor, and resistance to pests and diseases. The evaluation and selection locations were Lethbridge, Alberta (AB), St-Augustin-de-Desmaures, Quebec (QC), Tulelake, California (CA), Ithaca, New York (NY), and Prairie du Sac, Wisconsin (WI) (Supplementary Fig. 5b).

Additionally, four commercial cultivars were included for comparison: 55H94, AmeriStand 427TQ, Hybriforce-4400, and Vernal. Cultivars were selected from different breeding programs representing the diversity of alfalfa in North America. 55H94 check cultivar, developed by DuPont Pioneer in 2011, is highly resistant to potato leafhopper and offers high-quality forage, high yield, strong winter hardiness (WH = 8), and moderate fall dormancy (FD = 5). AmeriStand 427TQ, a high-yield, fast-recovery cultivar developed by America’s Alfalfa in 2014, has an FD of 4.3 and a winter hardiness of 1.8. Hybriforce-4400, a hybrid alfalfa cultivar from Dairyland Seed introduced in 2020, is the fourth generation of a commercial hybrid with improved yield potential, an FD of 4, and winter hardiness of 2. Vernal, an older public cultivar developed by the University of Wisconsin in 1953, is well-suited to northern climates, with good winter hardiness (FD = 2) and moderate regrowth after cutting. For simplicity, we refer to these four commercial cultivars as populations in this work, totaling 28 populations.

### DArTag genotyping

Two to three leaflets (~ 100 mg) were collected from individual plants for DNA extraction and genotyping. Approximately 44 individual plants from each of the 28 populations were sampled, for a total of 1,233 individual plants genotyped. Leaf tissues were sent to Intertek (Intertek; Alnarp, Sweden) for DNA extraction, the DNA samples were then sent to Diversity Arrays Technology Ltd. (DArT; Canberra, Australia)^52^ for genotyping using the alfalfa 3 K DArTag genotyping platform developed by Breeding Insight (Breeding Insight; Ithaca, NY, USA) of the USDA ARS^18^.

DArTag is an amplicon-based targeted genotyping platform developed by DArT^52^. The alfalfa DArTag platform consists of DNA oligo primers targeting genomic regions containing 3,000 SNP loci distributed across the alfalfa genome. DArTag probes hybridize to genomic DNA (gDNA) to capture the target SNP into the DArTag molecule. These DArTag molecules, along with unique barcodes for downstream demultiplexing, are then amplified. Subsequently, DArTag molecules with barcodes are purified and quantified before being sequenced on an Illumina platform (~ 100 × per marker per sample). DArT processed the sequencing reads and provided the Missing Allele Discovery Count (MADC) file.

MADC contains read depths for target and off-target SNPs allocated within the 3,000 genomic regions. Any short stretch of sequence with a unique combination of variants in MADC file is referred to as a unique microhaplotype^18^, where reference and alternative microhaplotypes only contain reference and alternative bases at the target SNP sites, respectively while RefMatch and AltMatch microhaplotypes contain off-target SNPs in addition to the reference and alternative allele at the target SNP sites, respectively.

### Filtering and allele dosage estimation

The alfalfa 3 K DArTag panel was developed based on the original 54-bp amplicon output; however, some amplicons were 81 bp in length. Therefore, adaptor sequences (ACCGATCTCGTATGCCGTCTTCTGCTTG) were removed using cutadapt v.4.8^53^. Filtering was then applied at the microhaplotype level, retaining RefMatch and AltMatch microhaplotypes if they met the following criteria: 1) they were present in at least 10 samples, with each sample having at least 2 detected reads; 2) they had > 90% identity and > 90% coverage of the reference microhaplotypes. Samples with ≥ 95% missing data were excluded. Subsequently, filtering at the marker loci was conducted, requiring ≥ 10 samples, with each sample having ≥ 10 reads for each marker locus. For marker loci with ten or more microhaplotypes, only the reference and alternative microhaplotypes were retained, as the excess of RefMatch and AltMatch microhaplotypes were likely due to paralogous amplification rather than true target loci amplification.

The filtered MADC with genomic regions and observed read counts was imported into R using the `readDArTag` function to create RADdata objects using the polyRAD v.2.0.0 R package^54^. Data overdispersion was determined using the `TestOverdispersion` function in polyRAD and applied to genotype estimation through the `IteratePopStruct` function, which allowed genotype calling, including for missing data. The final genotype calls at microhaplotype level were exported as a Variant Call Format (VCF) file using the `RADdata2VCF` function with the option asSNPs = False. Genotype dosages of the 3 K target SNPs were called following similar steps that performed for microhaplotype, but only used read depths of reference and alternative microhaplotypes from the filtered MADC.

### Genetic diversity

Genetic diversity indices were calculated for microhaplotype and target SNPs using the software GenoDive v.3.0^55^. Intrapopulation genetic diversity metrics were number of alleles (Num), effective number of alleles (N_E_), observed heterozygosity (H_O_), expected heterozygosity within population (H_S_), percentage of monomorphic loci (%mon), inbreeding coefficient (F_IS_), polymorphism information content (PIC), and total heterozygosity (H_T_).

Several interpopulation genetic diversity indexes were calculated, including F_ST_, Rho, and Analysis of Molecular Variance (AMOVA). The F_ST_ or Wright’s fixation index is the degree of genetic differentiation among populations in terms of allele frequencies^34^. F_ST_ is based on the idea that populations that are not intermating have different allele frequencies and can be calculated as

1 $${F}_{ST}=\frac{{H}_{T}-{H}_{S}}{{H}_{T}}$$

where H_T_ and H_S_ are the total and expected heterozygosity, respectively. F_ST_ range from 0 to 1, where 0 is no genetic divergency and 1 is fixation for alternative allele between populations. Genetic differentiation can be small (0 to 0.05), moderate (0.05 to 0.15), large (0.15 to 0.25) or very large (> 0.25). Ronfort et al. (1998) adapted F_ST_ to estimate population structure in autotetraploid species by including ploidy level (*k*) in the computation of the parameter Rho $(\rho )$ as follows:

2 $$\rho =\frac{{H}_{T}-{H}_{S}}{{H}_{T}-({H}_{O}\frac{\left(k-1\right)}{k})}$$

$\rho$ can be used to assess population structure across many loci, comparable between ploidy levels, and is independent of double reduction^35^. F_ST_ and $\rho$ range from 0 to 1, where 0 indicates no genetic divergence and 1 indicates fixation of alternative alleles between populations. Genetic differentiation can be small (0 to 0.05), moderate (0.05 to 0.15), large (0.15 to 0.25), or very large (> 0.25). To partition the genetic marker diversity among and within populations, we used AMOVA adapted to autopolyploids^56,57^. F_ST_, $\rho$, and AMOVA were computed using the software GenoDive v.3.0^55^.

### Population structure

Population structure analysis was performed using Principal Component Analysis (PCA) and Discriminant Analysis of Principal Components (DAPC) approaches^45^. PCA was performed for both target SNPs and microhaplotype markers using the software GenoDive v.3.0^55^. Only target SNPs were used to evaluate genetic population clustering by DAPC using adegenet R package v.2.1.10^45^ because the software only accepts biallelic markers. Marker matrix, marker position, population, and plant IDs were converted into a `genlight` object. DAPC was used to define clusters (k) of related genotypes without a priori information using multiple principal components to obtain a preliminary data transformation for discriminant analysis (DA). The function `find.clusters` was used to pre-define groups with the following paraments: method = Ward’s, criterion = goodfit, stat = Bayesian Information Criterion, and k = 5. The function `dapc` identified the genotypes group membership with the `find.clusters` function, keeping 80% of total variance retained.

### Linkage disequilibrium

Linkage disequilibrium (LD) is the nonrandom association of alleles at two or more loci in a population. LD decay was calculated using target SNPs with the R package ldsep v.2.1.5^58^. LD decay was estimated using squared allele-frequency correlations (${r}^{2}$). The SNPs with minor allele frequency lower than 5% were excluded in the estimates of LD. The rate of LD decay was estimated using ${r}^{2}$ and the distances in base pairs (bp) based on the *M. sativa* cultivar XinJiangDaYe reference genome using a nonlinear model previously used in different studies^48,50,59^. The expected ${r}^{2}$ value under drift-recombination equilibrium and low level of mutation was described in the appendix 2 of Hill and Weir, 1988^60^ as

3 $$E\left({r}^{2}\right)=\left[\frac{10+C}{(2+C)(11+C)}\right]\left[1+\frac{(3+C)(12+4C+{C}^{2})}{n(2+C(11+C))}\right]$$

where $C=4ad$, where $a$ is an estimated regression coefficient and $d$ is the physical distance in base pairs.

## Electronic supplementary material

Below is the link to the electronic supplementary material.

Supplementary Material 1

## Data Availability

The AMCC, MADC, genotypic matrix in GenoDive format, R script, and metadata files available in figshare (10.6084/m9.figshare.25537675.v1).
