# Author Correction: Pre-breeding in alfalfa germplasm develops highly differentiated populations, as revealed by genome-wide microhaplotype markers

**DOI:** 10.1038/s41598-025-94436-w

**Published:** 2025-04-08

**Authors:** Cesar A. Medina, Dongyan Zhao, Meng Lin, Manoj Sapkota, Alexander M. Sandercock, Craig T. Beil, Moira J. Sheehan, Brian M. Irish, Long-Xi Yu, Hari Poudel, Annie Claessens, Virginia Moore, Jamie Crawford, Julie Hansen, Donald Viands, Michael D. Peel, Neal Tilhou, Heathcliffe Riday, E. Charles Brummer, Zhanyou Xu

**Affiliations:** 1 Plant Science Research Unit, USDA-ARS, St. Paul, MN USA; 2 Breeding Insight, Cornell University, Ithaca, NY USA; 3 Plant Germplasm Introduction and Testing Research Unit, USDA-ARS, Prosser, WA USA; 4 Lethbridge Research and Development Center, Agriculture and Agri-Food Canada, Lethbridge, AB Canada; 5 Quebec Research and Development Centre, Agriculture and Agri-Food Canada, Québec, QC Canada; 6 School of Integrative Plant Science, Plant Breeding and Genetics Section, Cornell University, Ithaca, NY USA; 7 Forage and Range Research Unit, USDA-ARS, Logan, UT USA; 8 Dairy Forage Research Center, USDA-ARS, Madison, WI, US USA; 9 Department of Plant Sciences, University of California Davis, Davis, CA USA

Correction to: *Scientific Reports* 10.1038/s41598-024-84262-x, published online 08 January 2025

The Acknowledgments section in the original version of this Article was omitted. The Acknowledgments section now reads:

“Partial funding for this research was provided through NIFA Award Number 2019-70005-30361 from the NIFA-ASAFS Program to ECB, HR, DV/VM, and BI. This project is part of the NE-2210 (formerly NE-1710) Hatch Multistate Research Project.”

The original Article has been corrected.

