# Supplementary material for: Pre-breeding in alfalfa germplasm develops highly differentiated populations, as revealed by genome-wide microhaplotype markers
Source: Sci Rep. 2025 Jan 8;15:1253. doi: 10.1038/s41598-024-84262-x (PMC11711157; doi:10.1038/s41598-024-84262-x)
Supplement: Supplementary file 1 — Supplementary Material 1 [file 41598_2024_84262_MOESM1_ESM.docx]

**Supplementary Figures**

**
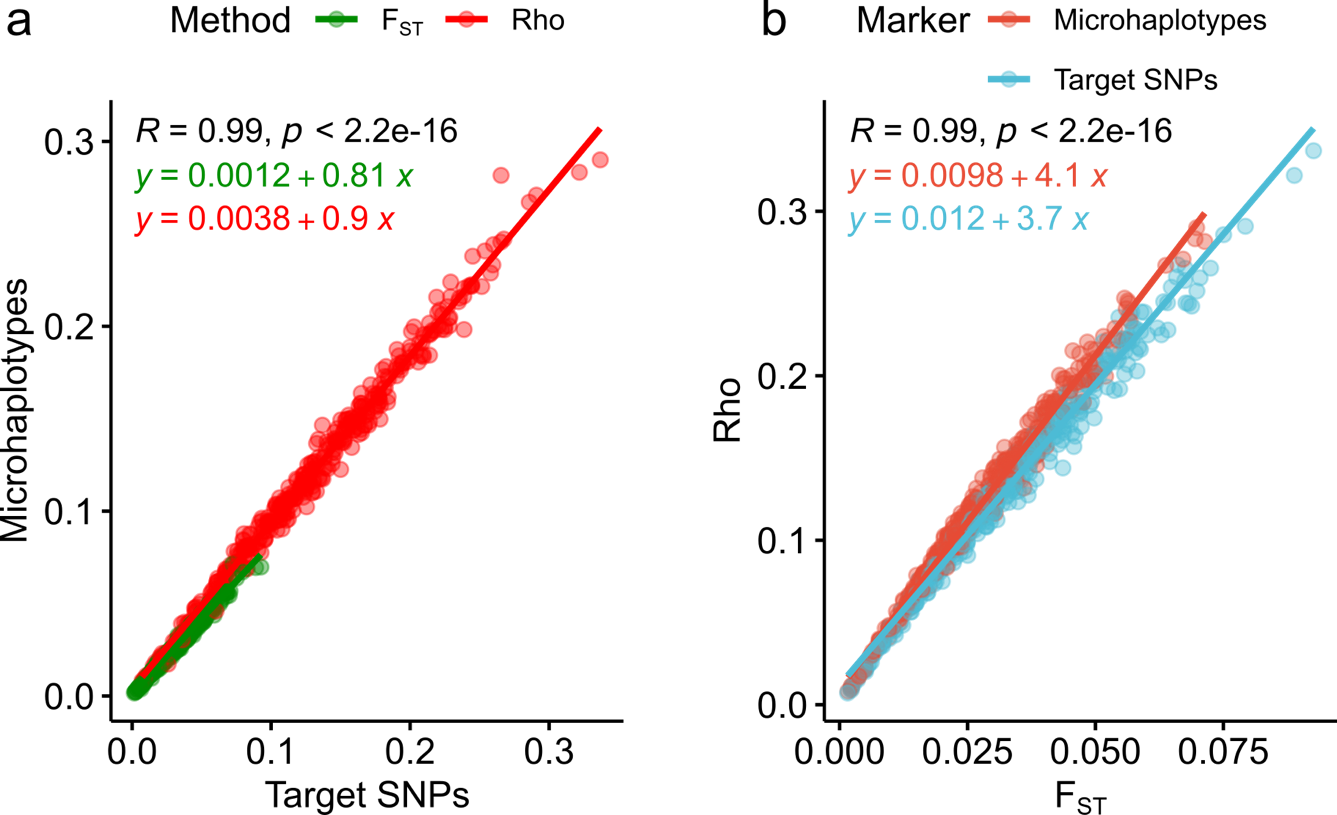
**

**Supplementary Figure 1.** **Comparison of interpopulation diversity parameters** **between Target SNPs and microhaplotypes.** **a.** Linear regression between of F_ST_ (green) and Rho (red) pairwise values in target SNPs and microhaplotypes. **b.** Linear regression between of target SNPs (Blue) and microhaplotypes (Red) pairwise values using F_ST_ and Rho parameters. Pearson’s correlation (R) and p value was the same for all regressions.


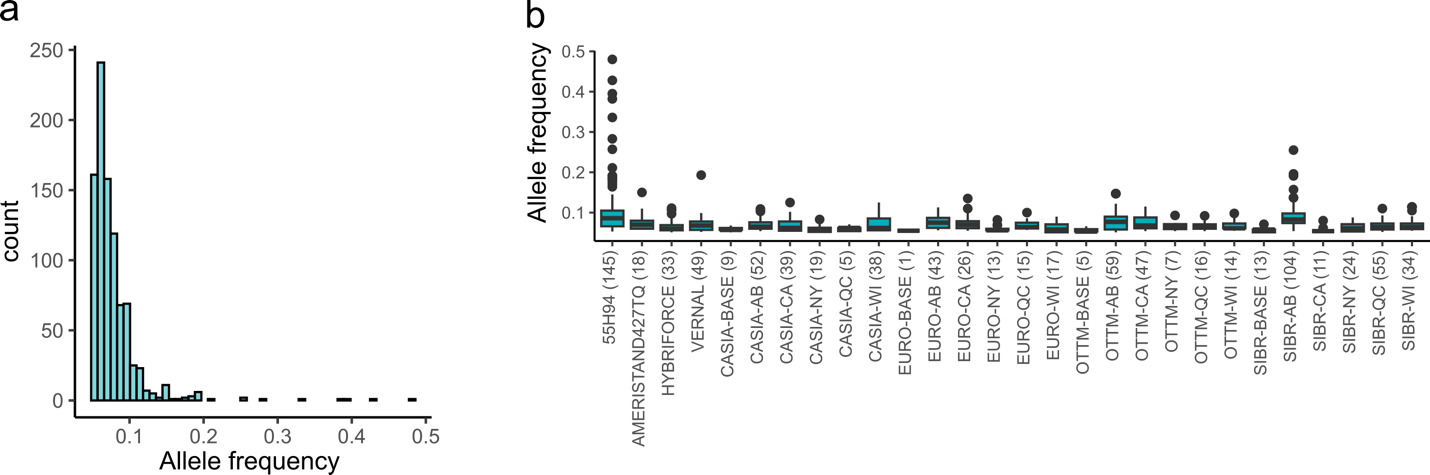


**Supplementary Figure 2.** **Private alleles in microhaplotypes.** **a.** Distribution of frequencies of private alleles (with frequency > 5%) in microhaplotypes. **b.** Boxplot of private alleles by each population. Number in brackets indicates the total of private alleles by population.


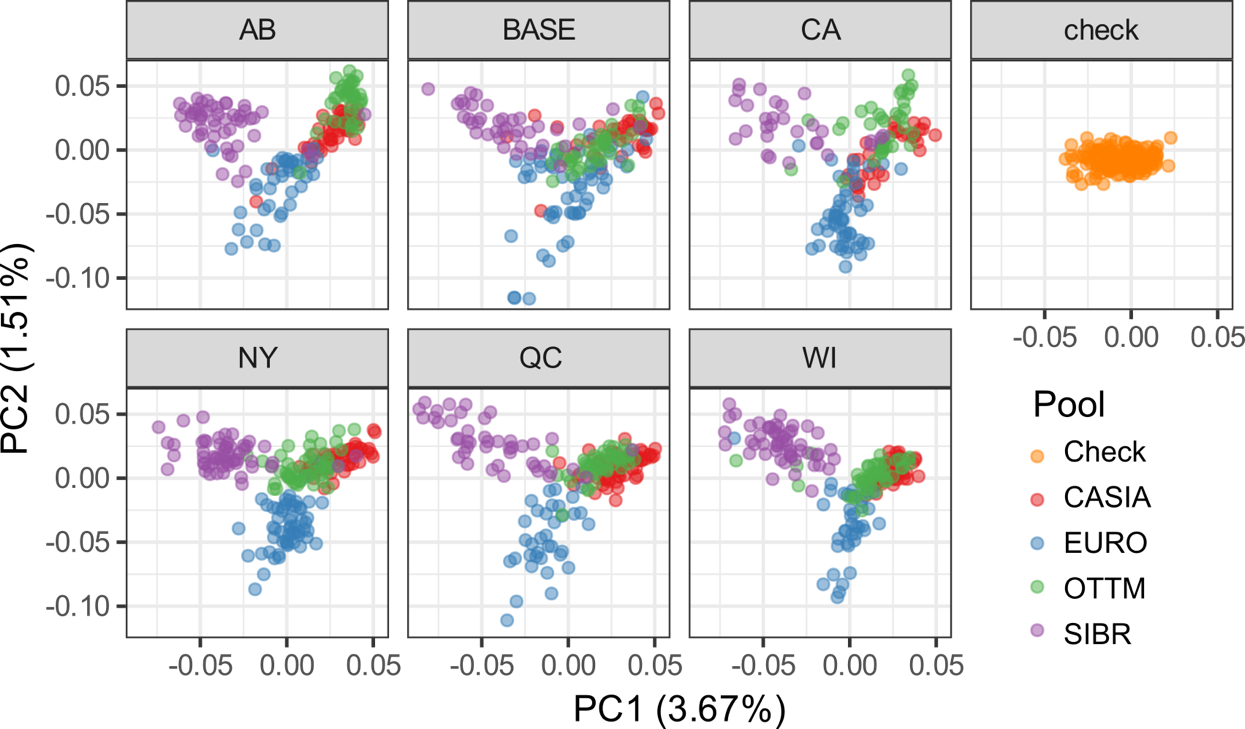


**Supplementary Figure 3. Two-dimensional scatter plot from principal component analysis by locations.** 1,243 accessions genotyped with target SNPs grouped by check cultivars, base populations, or locations: Lethbridge, Alberta (AB), St-Augustin-de-Desmaures, Quebec (QC), Tulelake, California (CA), Ithaca, New York (NY), and Prairie du Sac, Wisconsin (WI).


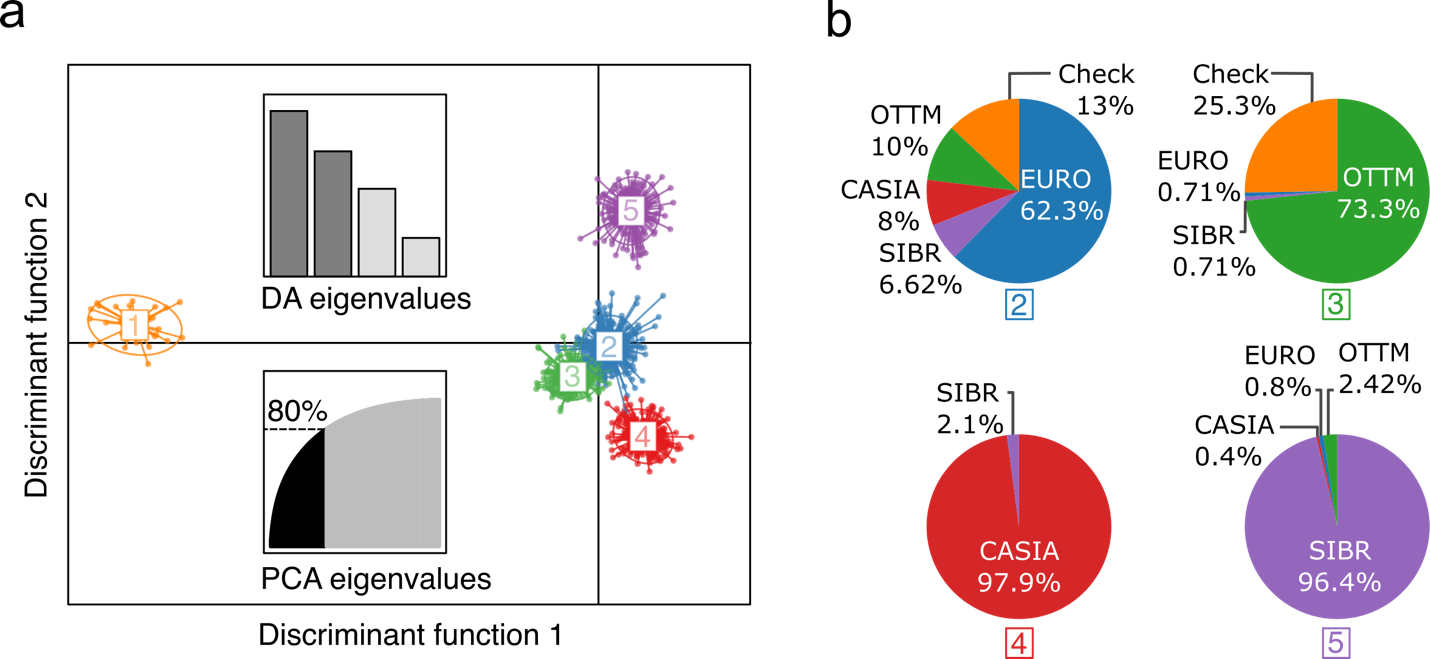


**Supplementary Figure 4. Population Structure by Discriminant Analysis of Principal Component (DAPC). a.** DAPC scatter plot for 1,243 alfalfa accessions using target SNPs. The axes represent the first two linear discriminants. Each oval represents a numbered cluster, and each dot represents a genotype. The inset charts indicate the number of principal components retained by DAPC (upper bar plot) and the cumulative variation retained during the analysis to describe the relationship between the clusters (lower inset). **b.** Pie chart of the percentage of pools in clusters generated by DAPC. Cluster one was composed only of the check variety 55H94 and was not included.


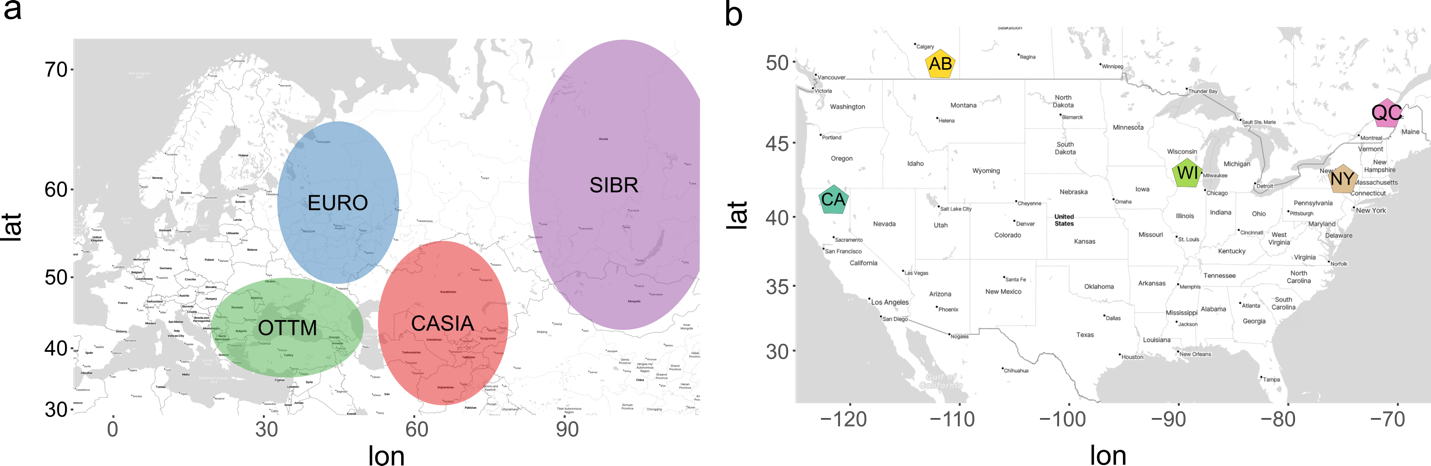


**Supplementary Figure 5. Geographical locations of germplasm pools used in this study a.** BASE populations were generated by intermating germplasm pools from four defined regions: Central Asia (CASIA), northeastern Europe (EURO), Balkans-Turkey-Black Sea (Ottoman) (OTTM), and Siberia/Mongolia (SIBR) regions **b.** 20 Cycle 1 (C1) populations were independently developed in five different locations: Lethbridge, Alberta (AB), St-Augustin-de-Desmaures, Quebec (QC), Tulelake, California (CA), Ithaca, New York (NY), and Prairie du Sac, Wisconsin (WI).

**Supplementary Tables**

**Supplementary Table 1.** Intra-population genetic variability in pools of populations.

Values were calculated for pools of check cultivars (Check), BASE, and cycle one (C1) populations. Heterozygosity-based statistics are population size (size), number of alleles (Num), effective number of alleles (N_E_), observed heterozygosity (H_O_), subpopulation heterozygosity or gene diversity (H_S_), and total heterozygosity (H_T_). Size corresponds to the number of plants genotyped.

| **Pool** | **size** | **Num** | **N_E_** | **H_O_** | **H_S_** | **H_T_** |
| --- | --- | --- | --- | --- | --- | --- |
| *Target SNPs* | |  |  |  |  |  |
| Check | 165 | 1.915 | 1.413 | 0.259 | 0.247 | 0.252 |
| BASE | 176 | 1.924 | 1.423 | 0.257 | 0.252 | 0.256 |
| C1 | 892 | 1.977 | 1.408 | 0.257 | 0.247 | 0.255 |
| *Microhaplotypes* | |  |  |  |  |  |
| Check | 165 | 3.552 | 1.773 | 0.405 | 0.38 | 0.386 |
| BASE | 176 | 3.787 | 1.787 | 0.402 | 0.384 | 0.389 |
| C1 | 892 | 4.068 | 1.764 | 0.403 | 0.378 | 0.389 |

**Supplementary Table 2.** Pairwise F_ST_ values among 28 alfalfa populations calculated with target SNPs (upper diagonal) or with microhaplotypes (lower diagonal). The color scale starts from 0 (blue) low populations differentiation to 1 (red) high population differentiation. All F_ST_ values were significant at p-value < 0.001.

**Supplementary Table 3.** Pairwise Rho values among 28 alfalfa populations calculated using target SNPs (upper diagonal) and microhaplotypes (lower diagonal). The color scale starts from 0 (blue) low populations differentiation to 1 (red) high population differentiation. All Rho values were significant at p-value < 0.001.

**Supplementary Table 4.** Allele frequency of loci with F_ST_ values > 0.20. The allele frequency of microhaplotypes was calculated by population. Grey scale color was applied to visualize the differences in allele frequency by locus.

**Supplementary Table 5.** Population membership generated using Discriminant Analysis of Principal Component (DAPC).

| **Population** | **DAPC1** | **DAPC2** | **DAPC3** | **DAPC4** | **DAPC5** | **Total** |
| --- | --- | --- | --- | --- | --- | --- |
| 55H94 | 37 | 1 | − | − | − | 38 |
| AMERISTAND427TQ | − | − | 25 | − | − | 25 |
| HYBRIFORCE | − | 8 | 46 | − | − | 54 |
| VERNAL | − | 48 | − | − | − | 48 |
| CASIA-BASE | − | 4 | − | 40 | 1 | 45 |
| CASIA-AB | − | 6 | − | 42 | − | 48 |
| CASIA-CA | − | 17 | − | 15 | − | 32 |
| CASIA-NY | − | 2 | − | 46 | − | 48 |
| CASIA-QC | − | 4 | − | 53 | − | 57 |
| CASIA-WI | − | 2 | − | 38 | − | 40 |
| EURO-BASE | − | 54 | 1 | − | − | 55 |
| EURO-AB | − | 39 | − | − | 1 | 40 |
| EURO-CA | − | 50 | − | − | − | 50 |
| EURO-NY | − | 57 | 1 | − | − | 58 |
| EURO-QC | − | 35 | − | − | − | 35 |
| EURO-WI | − | 38 | − | − | 1 | 39 |
| OTTM-BASE | − | 6 | 32 | − | − | 38 |
| OTTM-AB | − | 2 | 37 | − | − | 39 |
| OTTM-CA | − | 3 | 33 | − | 1 | 37 |
| OTTM-NY | − | 9 | 41 | − | 1 | 51 |
| OTTM-QC | − | 17 | 29 | − | − | 46 |
| OTTM-WI | − | 7 | 34 | − | 4 | 45 |
| SIBR-BASE | − | 4 | − | 1 | 34 | 39 |
| SIBR-AB | − | 1 | 1 | 1 | 48 | 51 |
| SIBR-CA | − | 7 | − | 1 | 21 | 29 |
| SIBR-NY | − | 6 | − | 2 | 49 | 57 |
| SIBR-QC | − | 4 | 1 | − | 39 | 44 |
| SIBR-WI | − | 7 | − | − | 48 | 55 |
| Total | 37 | 438 | 281 | 239 | 248 | 1,243 |
